# Molecular Characterization of ‘*Candidatus* Liberibacter Asiaticus’ Strains from Commercial Citrus-Growing Regions in Cuba Using Polymorphic Regions

**DOI:** 10.3390/microorganisms13102381

**Published:** 2025-10-15

**Authors:** Camilo Paredes-Tomás, Maritza Luis-Pantoja, Miguel Ramos-Leal, Marialea Melle, Assunta Bertaccini

**Affiliations:** 1 Instituto de Investigaciones en Fruticultura Tropical, Miramar, La Habana 11300, Cuba; maritzaluispantoja@gmail.com (M.L.-P.); mramosleal5@gmail.com (M.R.-L.); 2 Department of Agricultural and Food Sciences, *Alma Mater Studiorum*—University of Bologna, 40127 Bologna, Italy; leamelle9@gmail.com

**Keywords:** citrus greening, “huanglongbing”, Cuban strains, citrus orchards

## Abstract

Studies of the genetic diversity of ‘*Candidatus* Liberibacter asiaticus’ strains based on housekeeping genes have been unsuccessful. The increasing availability of complete genome sequences of several strains from different countries has allowed the identification of regions having greater variability, which have been successfully implemented for the bacterium characterization, including microsatellites, genes of prophage origin, and miniature transposable elements with inverted-repeats (MITEs). In the present work, the genetic structure of 147 ‘*Ca*. L. asiaticus’ strains from nine provinces of Cuba were investigated using two polymorphic regions, consisting of typing for prophages and MITEs. The results showed an important level of coexistence of type 1 and 2 prophages in the Cuban strains, while the type 3 prophage was not detected. Likewise, a high rate of co-occurrence of both types of MITEs (MCLas-A and -B) was also observed. However, the MITE MCLas-A was detected only in its empty form. The double-locus analysis allowed the identification of eight genotypes. Out of these, seven genotypes were present in the Western region, which constitutes the region with the highest genetic variability. This is the first report of a genetic characterization of Cuban strains of ‘*Ca*. L. asiaticus’ with polymorphic markers in orchards growing in commercial citrus regions.

## 1. Introduction

“Huanglongbing” (HLB), also known as citrus greening, is the most threatening disease in citrus farming worldwide [1]. Its occurrence has been associated with the presence of three bacteria within the ‘*Candidatus* Liberibacter’ genus: ‘*Ca*. L. asiaticus’, ‘*Ca*. L. africanus’ and ‘*Ca.* L. americanus’ [2]. ‘*Ca*. L. africanus’ is located in Africa, where it is transmitted by *Trioza erytreae*, while ‘*Ca*. L. asiaticus’ and ‘*Ca*. L. americanus’ are both transmitted by *Diaphorina citri* in Asia, and the latter is transmitted in America [3]. The most widespread pathogen is ‘*Ca*. L. asiaticus’, endemic in Asia but detected as epidemic in the other geographic areas. Identifying and characterizing the metabolic pathways to support the infection cycle in the plant and insect vector hosts in this pathosystem has been hindered by the inability to establish these fastidious bacteria in pure culture; however, genomic analyses have revealed the presence in this bacterium of genes coding secretion systems, motility structures, *quorum-sensing* components, and prophage-like elements [4,5].

The presence of *D. citri* was reported in Cuba in 1999 [6]. However, the first symptomatic plants were detected at the end of 2006 [7]. The genetic diversity studies using housekeeping genes (i.e., the 16S rRNA and the intergenic region 16S-23S) [8,9,10], the *omp* gene [11], or the *operon β* genes [12] have allowed the differentiation of the three ‘*Candidatus* Liberibacter’ species [8,9,11]. Based on these regions, ‘*Ca*. L. asiaticus’ is the only species detected to date in Cuba [7,13]. However, the highly conserved nature of these genes limits the discriminatory power for more closely related bacteria (i.e., strains) [14]. Recently, genomic regions with greater variability have been successfully implemented, including microsatellites [14,15,16], three types of prophages [17,18,19], and two types of miniature inverted-repeat transposable elements (MITEs) (i.e., MITE MCLas-A and MITE MCLas-B) [20] identified inside the genome of these bacteria. Regarding this last marker, DNA bands of three different lengths were detected. The B630 and B350 (representing the filled and empty MITE-A, respectively) and B720 (representing the filled form of MITE MCLas-B) are all differentiable by their sizes. The multilocus analysis has been proven to be a better approach, allowing an improved genetic differentiation tool for these and other insect-transmitted bacteria [20,21,22,23].

Due to their greater variability, polymorphic regions are suitable tools to evaluate genetic variability within populations of the same species. In Cuba, previous strain differentiation works based on the study of the conserved regions were unsuccessful [7,13]. Recently, the combined use of prophage types and MITEs allowed the differentiation of six strains of ‘*Ca*. L. asiaticus’ based on the length of amplified bands [23]. However, these tools have been successfully used before [23] on a small scale; therefore, extensive use of these tools opens a new approach for the molecular epidemiology of HLB. Similar strategies have been proven essential for understanding the introduction events of both the insect vector [24,25] and the circulating strains of the bacterium [15,26].

The objective of this work was to investigate the genetic diversity of 147 ‘*Ca*. L. asiaticus’ strains collected in commercial orchards from nine Cuban provinces using two genetic markers, including type-specific prophage *loci* and a miniature inverted-repeat transposable elements (MITEs) region.

## 2. Materials and Methods

### 2.1. Plant Material and DNA Extraction

**Figure 1:**
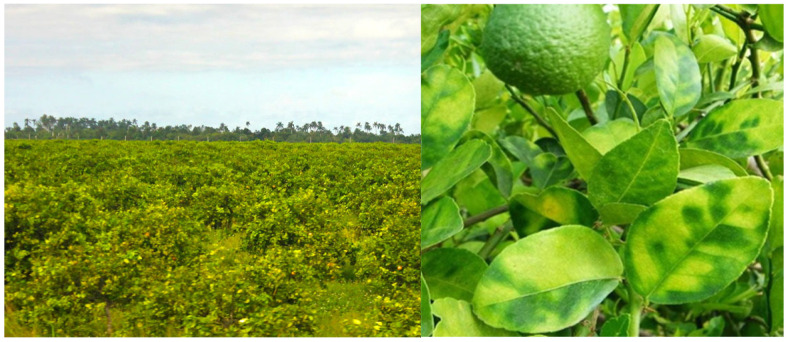
Citrus orchard inspected during the samplings, and the blotchy mottle as the main characteristic symptom associated with HLB presence.

**Figure 2:**
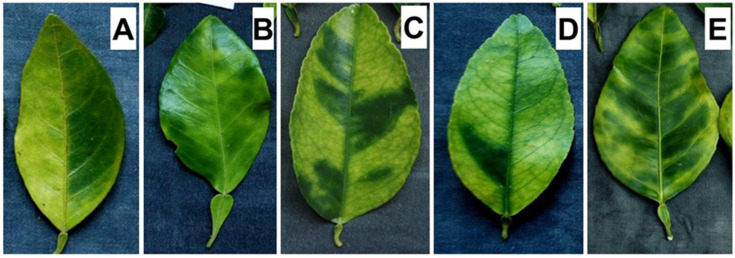
The asymmetric blotchy mottle, as the main characteristic symptom associated with HLB presence in sweet orange (**A**), grapefruit (**B**), Persian lime (**C**), Mexican lime (**D**), and sour orange (**E**).

A total of 168 symptomatic citrus samples (Figure 1) were collected in nine provinces of Cuba during extensive samplings conducted in the period 2014–2020. Each sample consisted of 10–15 leaves per tree. HLB-associated symptoms include yellow shoots, asymmetric blotchy mottle, yellow and corking veins, nutrient lack symptoms, and small leaves [27]. Of these, the asymmetric mottle is the most characteristic symptom in all the cultivated citrus species in Cuba. Additionally, it has shown the highest diagnostic value compared to the other symptoms, making it ideal for field diagnosis. The samples included mature symptomatic leaves showing the mottle and preferably without damage from other pests (e.g., citrus leaf miner) that could hinder accurate recognition. Likewise, the different manifestations across the various sampled citrus species were taken into account (Figure 2). These samples were representative of the main geographic regions of the country, i.e., Western, Central, and Eastern (Table 1).

The sampled citrus plants included Persian lime (*Citrus latifolia* Tan.), grapefruit (*Citrus paradisi* Macf.), sweet orange [*Citrus sinensis* (L.) Osb.], Eureka lemon [*Citrus limon* (L.) Burm. f.], Mexican lime [*Citrus aurantifolia* (Chrism.) Swing.], and sour orange (*Citrus aurantium* L.). The age of the trees varied from 4 to 6 years. The leaf samples were collected mainly based on the presence of asymmetric blotchy mottle, a symptom with the greatest diagnostic value associated with ‘*Ca*. L. asiaticus’ infection under environmental Cuban conditions [26]. Symptomatic citrus plants infected with a ‘*Ca*. L. asiaticus’ strain previously characterized were used as positive amplification control [23]. Asymptomatic citrus plants, maintained under protected greenhouses, were used as negative controls. A total of 10–15 mature leaves per tree were collected, and DNA extraction was performed from 0.6 g of leaf midribs using CTAB [28] method. The final pellet was diluted in 100 µL of free-RNA water. A total of 168 plants were collected for the analysis, and the bacterial DNA extracted from symptomatic leaves of one individual tree represented one strain of ‘*Ca*. L. asiaticus’; analyses were repeated at least twice for each sample.

### 2.2. PCR Assay for Bacteria Detection

The presence/absence of ‘*Ca*. L. asiaticus’ was verified using a nested PCR assay combining the universal and specific primers fD1/rP1 [29] and OI1/OI2c [30] (Table 2). PCR assays with the last pair of primers (i.e., OI1/OI2c) were used to select the samples for subsequent analysis. For the prophage typing analyses, the following pairs of primers were used for PCR assays: T1-1F/T1-1R (type 1 prophage), T2-1F/T2-1R and T2-8F/T2-8R (type 2 prophage), and 891-1F/891-1R (type 3 prophage) [19,20]. For the MITEs detection, the primer pair LapPF1-F/LapPF1-R [20] was used. All the reactions were performed according to previously optimized procedures [23]. Aliquots of all the amplified DNAs (6 µL) were subjected to electrophoresis in 2% agarose gel (1X Tris-acetate/EDTA), stained with ethidium bromide, and observed under ultraviolet light in a transilluminator.

## 3. Results

**Figure 3:**
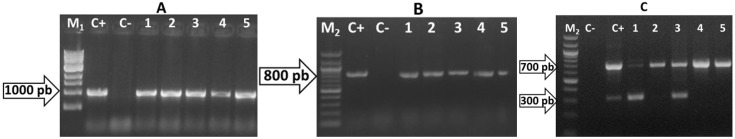
Results of amplification of polymorphic regions from ‘*Ca*. L. asiaticus’-Cuban strains from *Citrus* spp. in 2% agarose gel stained with ethidium bromide. (**A**) Prophage type 1, (**B**) prophage type 2, and (**C**) predominant profiles of MITEs. Lanes 1: sample 2680; lanes 2: sample A01320; lanes 3: sample 2616; lanes 4: sample 2657; lanes 5: sample A00315; C+: citrus sample LPIJ4 [23]; C−: citrus sample 2345; M1: 1 kb DNA ladder (BioLabs, Boston, MA, USA); and M2: 100 bp DNA ladder (BioLabs, USA).

During the samplings, symptomatic plants were observed in all the provinces where citrus are grown. The most common symptom was the asymmetric leaf mottle (Figure 1). The microscopic symptoms associated with the disease include excessive callose accumulation, which causes obstruction of the phloem tubes and leads to the interruption of the phloemic transport. As a consequence, the abnormal accumulation of photosynthates in leaves induces thylakoids and chlorophyll degradation. All these changes result in a reduction in photosynthetic area as well as a functional disconnection between the sink and source tissues in the plant. The presence of ‘*Ca*. L. asiaticus’ was confirmed in 165/168 (98%) of the samples with nested PCR assay. For subsequent analyses, the samples with bacterial presence detectable by PCR assay were selected among those amplified with primers OI1/OI2c. This allowed the selection of 147 out of the 168 samples (88%, including strains from all provinces and citrus types involved in the sampling) in which a band of the expected size (1160 bp) was obtained (Table 1). The PCR assays for the prophage types allowed the detection of two out of the three known types (i.e., prophage type 1 or 2 or both coexisting) in the 147 strains of ‘*Ca*. L. asiaticus’ (Table 3). The type 3 prophage was not detected at all. Almost all the strains (143/147; 97.3%) resulted in amplification with PCR markers specific for prophage types 1 and 2, indicating a high rate of coexistence of both prophages in the same strains (Figure 2). This tendency was predominantly observed regardless of the citrus type or the geographic region. Only in four strains (4/147; 2.7%), three from the western region and one from the eastern region, was only one of the two prophage types detected (prophage type 1 or 2 individually). No strains with an absence of prophage were detected (Table 3 and Figure 3).

Based on the primers used for the detection of the two MITEs (i.e., MCLas-A and -B) proposed by Wang et al. [20], four types of patterns were identified in the 147 samples: B720, B350, B720 + B350, and no bands (Table 4). The type B720 + B350 (i.e., high rate of coexistence of both MITEs) was the predominant genotype (105/147; 71.4%) being detected in all the nine provinces surveyed, followed by strains carrying the MITE CLas-B (B720) (32/147; 21.8%) and the empty MITE CLas-A (B350) (7/147; 4.8%).

Using the information from both *loci* obtained in the present study, 8 out of the 12 possible double-*locus* genotypes (DL genotypes) were identified within the 147 analyzed strains (Table 5). The DL-12 genotype (corresponding to B720 + B350 with prophage types 1 and 2), followed by the DL-9 genotype (B720 with both prophage types), was present in 103 (70,1%) and 32 (21,8%) of the samples, respectively. The DL12 genotype was detected in all the citrus species. After grouping the strains by the double *loci* according to the geographical origins (i.e., western, central, and eastern regions), the western region showed the largest strain variability, containing 7 of the 8 DL genotypes identified in this analysis, while the DL-4 genotype was only detected in the eastern region.

## 4. Discussion

The results presented here confirm the widespread occurrence of HLB disease across Cuba, as well as the susceptibility of all the cultivated citrus species to the infection by ‘*Ca*. L. asiaticus’. These observations are consistent with previous findings in Cuba and elsewhere [30,31,32,33]. The fact that 165 out of 168 leaf samples showing the asymmetric blotching mottle symptom tested positive for ‘Ca. L. asiaticus’ is proof of the high diagnostic value of this specific symptom for the visual identification of the disease’s presence in the orchards [26].

In ‘*Ca*. L. asiaticus’ strain sequences the presence of a number of prophage regions were detected [34,35]. Prophages, the lysogenic form of a bacterial phage with its DNA inserted into the chromosome, are important genetic elements of the bacterial genome and play critical roles in bacterial evolution, bacterial cell defense, and environmental adaptation, including pathogenesis [36,37]. In particular, three types of prophages—type 1, represented by prophage SC1; type 2, represented by prophage SC2; and type 3, represented by prophage P-JXCG-3—have been described in detail [19,31,38]. SC1 is involved in the lytic cycle of forming phage particles, and SC2 in the lysogenic conversion of ‘*Ca*. L. asiaticus’ pathogenesis [38,39,40]. They are structurally divided into early gene and late gene regions. The early gene region is conserved and associated with DNA replication, and the late gene region is associated with genes encoding proteins involved in the lytic cycle [38]. Structures of the three prophages suggested a model consisting of a core (early genes) region, designated as C, and a flexible (late genes) region, designated as F, with the F possessing genes of different biological functions [19,34]. Out of these, only the type 1 and 2 prophages coexisted in almost all the Cuban samples tested. Da Silva et al. [41] also reported the large coexistence of both prophages in Brazilian strains of ‘*Ca*. L. asiaticus’ since they were detected in 289 out of 299 samples. The close phylogenetic relationships between Cuban and Brazilian ‘*Ca*. L. asiaticus’-strains were previously reported based on housekeeping gene sequences using the sequences of 16S, 16S/23S, and *rplKAJL* genes [32]. The similarities observed in the detection rates of prophage types in both bacterial populations constitute additional evidence supporting this hypothesis.

These population structures contrast strongly with what has been reported in provinces of southern China by Zheng et al. [19]. In the Asian populations, a strong segregation of prophages 1 or 2 was observed, as only a few of the analyzed samples (13/187; 6,95%) showed the copresence of the two bacterial types. Recently, Zheng et al. [42] corroborated the predominant prophage segregation in Chinese strains. They also found only a low number of strains (34 out of 819, 13.7%) carrying simultaneously prophage types 1 and 2. Assuming the infectious capacity of both phages, as well as the different genes identified in both genomes (genes involved in the insect acquisition, in the turn-off of the plant defenses, and for its integration into the host genome) proposed by Zhang et al. [38], a strain carrying both types of prophages, such as the Cuban ones, could have a greater adaptive advantage with respect to those carrying the single phages.

In regard to the MITE regions, Zheng et al. [42] reported the same four combinations of amplicons as those reported in the present study. Wang et al. [20] reported a high rate of detection of both MITEs in ‘*Ca*. L. asiaticus’ populations from Florida. However, almost all the strains showed both MCLas-A and MCLas-B filled (B630 and B720) and the empty prophage MCLas-A (B350). Only a few of the Florida strains were carrying the empty MCLas-A together with filled MCLas-B (B350 and B720, respectively), as it is detected in most of the Cuban strains. On the contrary, Wang et al. [18] and Zheng et al. [42] reported separation of MCLas-A and MCLas-B [31] in the Chinese populations. On the other hand, a few strains without MITEs were found in several Chinese provinces. However, this kind of situation was not observed in the Florida and Cuban strains.

This observation reinforces the hypothesis of the similarity of Cuban strains of ‘*Ca*. L. asiaticus’ with those circulating throughout the American continent. Additionally, the detection of the two states (i.e., empty and filled forms) of a transposon in a single strain constitutes evidence of its mobility. Based on this observation, MCLas-A was speculated to be an active transposon by Wang et al. [20]. The lack of evidence of MITE mobility in the Cuban population suggests a lack of transposition efficiency under Cuban conditions. The studies of the variability of ‘*Ca*. L. asiaticus’-strains based on a single *locus* may not reveal the large diversity inside of a population due to the difference in the evolutionary rate among different *loci.* Each *locus* may have its own evolutionary rate, which could be responsible for the formation of diversified genotypes [20]. The multilocus approach allowed for a more detailed genetic characterization and description of the Cuban ‘*Ca*. L. asiaticus’ populations with respect to what can be concluded by studying each *locus* individually. Two genotypes were preliminarily reported after testing six Cuban ‘*Ca*. L. asiaticus’ strains [23]. This result made it possible to determine the suitability of the polymorphic regions for a better characterization of Cuban strains within the local ‘*Ca*. L. asiaticus’ populations. The two identified genotypes were increased to eight with the same approach on a large number of samples. These observations indicate that the number of samples tested is a critical point for accurate population characterization.

The presence of the DL-12 genotype infecting all the citrus species suggests that the coexistence of both phages could constitute crucial elements in the adaptability of ‘*Ca*. L. asiaticus’ to different plant hosts. The western region, in which 7 out of the 8 genotypes were detected, was identified as the geographic location with the highest genetic variability. This, together with the detection of the first symptomatic plants in late 2006 in residential areas of Havana [7,43], constitutes the first scientific evidence of a possible first introduction to the island through this region. However, the detection of a double-*locus* genotype only happened in the eastern region (i.e., the DL-4 genotype), suggesting the possibility of more than one introductory event to the country. In contrast, the lowest variability was found among strains from the central region, mostly belonging to citrus orchards located in Ceballos, Ciego de Ávila province. This was the only citrus-growing area in Cuba where the management of HLB included the eradication of the symptomatic plants from the earliest stages of the epidemic.

The management of the disease in Cuba was suggested based on three critical points: (i) the use of certified healthy propagation material, (ii) the control of the vector population, and (iii) the eradication of symptomatic plants. Of these measures, the latter was having less acceptance among the majority of Cuba. Only the ‘’Ceballos’’ enterprise has consistently integrated these three actions into an integrated management system.

The epidemiological studies performed during the last decade on Cuban citrus orchards have shown that the immediate removal of symptomatic plants in young plantations (i.e., less than three years old), along with monitoring and managing insect vector populations, considerably reduces the incidence and severity of the disease in the long term [33]. Incidences in areas with integrated disease management (lower than 5%) are highly contrasting to those where the eradication was not implemented despite the control of vector populations (61–100%). In the present study, the lowest variability was found among strains from the central region (with respect to the western and eastern regions of Cuba), mostly belonging to citrus orchards located in Ceballos, Ciego de Ávila province. This is the same location where the management of HLB included the eradication of symptomatic plants from the earliest stages of the epidemic. These facts together suggested that the constant eradication of infected plants is an effective way to minimize the spread of the disease and the inoculum sources. This result supports the hypothesis put forward by Da Silva et al. [41] that the constant eradication of infected plants is an effective way to minimize the spread of the disease and, therefore, a possible cause of the limited genetic variation in the Brazilian strains. In the western region of Cuba, HLB management is mostly based on chemical control of the insect vector with occasional eradication of infected plants [32]. This could facilitate the spread and establishment of new genotypes, which are detectable by their larger variability. These findings suggest the need for the early eradication of symptomatic plants plus the control of the vector population as an integrated approach rather than a single type of intervention for successful disease management that can also be durable over time.

## 5. Conclusions

The presence of ‘*Ca*. L. asiaticus’ was detected in almost all the collected samples, which confirmed the widespread presence of HLB in the commercial orchards in Cuba. Type 1 and 2 prophages were identified for the first time coexisting as predominant genotypes in the analyzed strains from all the Cuban citrus-growing provinces, suggesting that they can represent an adaptive advantage of ‘*Ca*. L. asiaticus’ in the country. The analysis of the MITEs region showed a low dynamic of the transposons in the Cuban strains, as no evidence of mobility was found for both MClas-A and -B. The double *locus* analysis of the Cuban strains supported, for the first time, the introduction of ‘*Ca*. L. asiaticus’ into Cuba through the western region and suggested a possible second introduction through the eastern region. The lowest genetic variability in ‘*Ca*. L. asiaticus’ strains from the Central area suggest the positive influence of the eradication of symptomatic plants not only in the management of the disease but also in the establishment of new genotypes.

Altogether, the study demonstrates the usefulness of polymorphic regions, such as prophage types and MITEs, in revealing the diversity of ‘*Ca*. L. asiaticus’ strains in commercial citrus orchards, which will lead to a better understanding of the epidemiology of the bacteria in Cuba. The subsequent detections of HLB in Florida and Cuba, as well as the geographic proximity, led to the belief that ‘*Ca*. L. asiaticus’ could have been introduced into the country through the western region. However, there is no scientific evidence to support this hypothesis at the moment.

## Figures and Tables

**Table 1:** ‘*Ca*. L. asiaticus’ detection in commercial citrus species and geographic regions evaluated using a PCR assay with OI1/OI2c primers.

Region	Sample Location/Enterprise/Province	Citrus	Number of ‘*Ca.* L. asiaticus’ Positive Plants/Total
Western	21°53′05″ N 82°48′04″ W Agroindustrial JMO/Isla de la Juventud	Persian lime	3/3
	22°31′50.6″ N 81°10′34.4″ W Agroindustrial VG/Matanzas	Sweet orange	17/21
		Grapefruit	17/19
		Persian lime	9/9
		Eureka lemon	7/9
	22°8′39″ N 80°12′38″ W Cítricos “Arimao”/Cienfuegos	Sweet orange	8/10
		Grapefruit	9/9
Central	21°39′46.969″ N 77°41′14.860″ W Cítricos “Sola”/Camagüey	Sweet orange	5/5
		Grapefruit	3/4
		Persian lime	6/6
	21°55′57.491″ N 78°45′6.646″ W Agroindustrial “Ceballos”/Ciego de Ávila	Sweet orange	17/17
		Grapefruit	10/10
		Persian lime	12/16
Eastern	20°21′54.454″ N 76°25′46.134″ W Jiguaní/Granma	Sweet orange	4/4
		Grapefruit	1/2
		Persian lime	3/3
	20°53′56.957″ N 76°35′2.189″ W Jíquima/Holguín	Mexican lime	0/1
	20°57′25.160″ N 75°43′7.848″ W Banes/Holguín	Mexican lime	3/3
	20°17′57.1″ N 76°14′40.5″ W Cítricos “AL”/Santiago de Cuba	Sweet orange	3/3
		Grapefruit	5/6
	20°20′41.1″ N 74°29′36.5″ W Patio de casa de visitas/Guantánamo	Persian lime	0/2
	20°16′43.4″ N 74°27′52.9″ W CSS “IA”/Guantánamo	Sour orange	1/1
		Mexican lime	1/1
	20°17′44.4″ N 74°28′25.3″ W Finca de Pucho Suarez/Guantánamo	Sour orange	0/1
	20°21′6.359″ N 74°30′7.634″ W Bariguita/Guantánamo	Sour orange	3/3

**Table 2:** Sequences of the primers used for the ‘*Ca*. L. asiaticus’ detection and characterization.

Primer	Sequences 5′–3′	*Locus* Taget	Amplicon Type (bp)
fD1	AGAGTTTGATCCTGGCTCAG	16S	Single (1400)
rP1	ACGGTTACCTTGTTACGACTT		
OI1	GCGCGTATCCAATACGAGCGGCA	16S	Single (1160)
OI2c	GCCTCGCGACTTCGCAACCCAT		
T1-1F	ATCCTTTGACAGTGAGGCCA	SC1_gp030	Single (1025)
T1-1R	CTCGTGAGGTTCTTGAGGGT		
T2-1F	GCACCTCTCGCATACCAAAG	SC2_gp030	Single (807)
T2-1R	GTCGGTGGTTTTACTCGCAA		
T2-8F	CATAGCCCCTCCCTCAGTTC	SC2_gp240	Single (795)
T2-8R	GCGGGAGTCAAGATAACACC		
891-1F	CTGATCCTTTACCATGCCGC	PJXGC_08	Single (950)
891-1R	CAGCGAAACCGATCTTGAGG		
LapPF1-f	GCCACTTTGGGGTAGCAGTA	CLIBASIA_05620 to CLIBASIA_05625	Multiple (350 and/or 720)
LapPF1-r	AAAACTTTCGTCACGGCTTT		

**Table 3:** Distribution of the Cuban strains of ‘*Ca*. L. asiaticus’ detected in commercial citrus species with different combinations of prophage types.

Province	Citrus	Number of Samples	‘*Ca*. L. asiaticus’ Strains According to Prophage Combinations			
			Type 1	Type 2	Type 3	Types 1 + 2
Isla de la Juventud	Persian lime	3	0	0	0	3
Matanzas	Persian lime	9	0	0	0	9
	Grapefruit	17	0	0	0	17
	Sweet orange	17	1	1	0	15
	Eureka lemon	7	0	0	0	7
Cienfuegos	Grapefruit	9	0	0	0	9
	Sweet orange	8	0	1	0	7
Ciego de Ávila	Persian lime	12	0	0	0	12
	Grapefruit	10	0	0	0	10
	Sweet orange	17	0	0	0	17
Camagüey	Persian lime	6	0	0	0	6
	Grapefruit	3	0	0	0	3
	Sweet orange	5	0	0	0	5
Granma	Persian lime	3	0	0	0	3
	Grapefruit	1	0	0	0	1
	Sweet orange	4	0	0	0	4
Holguín	Mexican lime	3	0	0	0	3
Santiago de Cuba	Grapefruit	5	0	0	0	5
	Sweet orange	3	0	0	0	3
Guantánamo	Mexican lime	1	0	0	0	1
	Sour orange	4	1	0	0	3
Total		147	2	2	0	143

**Table 4:** Distribution of the amplicon types with primer Lap-PF1-f/r in ‘*Ca*. L. asiaticus’ strains in commercial citrus orchards in Cuba.

Province	Citrus	Number of Samples	Numbers of ‘*Ca*. L. asiaticus’ Strains in Each Electrophoretic Band Type			
			B350	B720	B350 + B720	No Band
Isla de la Juventud	Persian lime	3	0	0	3	0
Matanzas	Persian lime	9	0	2	7	0
	Grapefruit	17	0	3	14	0
	Sweet orange	17	1	4	11	1
	Eureka limon	7	0	2	4	1
Cienfuegos	Grapefruit	9	0	3	6	0
	Sweet orange	8	1	2	5	0
Ciego de Ávila	Persian lime	12	1	1	10	0
	Grapefruit	10	0	2	8	0
	Sweet orange	17	0	8	9	0
Camagüey	Persian lime	6	0	0	6	0
	Grapefruit	3	0	0	3	0
	Sweet orange	5	0	0	5	0
Granma	Persian lime	3	0	1	2	0
	Grapefruit	1	0	0	0	1
	Sweet orange	4	0	3	1	0
Holguín	Mexican lime	3	0	1	2	0
Santiago de Cuba	Grapefruit	5	0	0	5	0
	Sweet orange	3	1	0	2	0
Guantánamo	Mexican lime	1	0	0	1	0
	Sour orange	4	3	0	1	0
Total		147	7	32	105	3

**Table 5:** Double *locus* (DL) analysis of both regions (prophage and MITE types) of the Cuban ‘*Ca*. L. asiaticus’ strains. The strains are grouped according to their geographic origin. Light grey background to highlight prophage presence.

				Prophage	
			**T1**	**T2**	**T1 + T2**
**MITEs**		Western	0	1	1
	No band	Central	0	0	0
		Eastern	0	0	1
			**DL-1**	**DL-2**	**DL-3**
				
		Western	0	0	2
	B350	Central	0	0	1
		Eastern	1	0	3
			**DL-4**	**DL-5**	**DL-6**
				
		Western	0	0	16
	B720	Central	0	0	11
		Eastern	0	0	5
			**DL-7**	**DL-8**	**DL-9**
				
		Western	1	1	48
	B720 + B350	Central	0	0	41
		Eastern	0	0	14
			**DL-10**	**DL-11**	**DL-12**

## Data Availability

The original contributions presented in this study are included in the article. Further inquiries can be directed at the corresponding authors.

## Abbreviations

The following abbreviations are used in this manuscript:

‘*Ca*. L. asiaticus’	‘*Candidatus* Liberibacter asiaticus’
CTAB	Hexadecyltrimethylammonium bromide
DL	Double locus
EDTA	Ethylenediaminetetraacetic acid
HLB	“Huanglongbing”
MITEs	Miniature inverted-repeat transposable elements
PCR	Polymerase Chain Reaction
